# Predictors of incomplete immunization coverage among one to five years old children in Togo

**DOI:** 10.1186/s12889-016-3625-5

**Published:** 2016-09-13

**Authors:** Dadja Essoya Landoh, Farihétou Ouro-kavalah, Issifou Yaya, Anna-Lea Kahn, Peter Wasswa, Anani Lacle, Danladi Ibrahim Nassoury, Sheba Nakacubo Gitta, Abdramane Bassiahi Soura

**Affiliations:** 1 World Health Organization, Country office, Togo, Lomé, Togo; 2 Division de l’épidémiologie, Ministère de la Santé du Togo, Lomé, Togo; 3 Aix-Marseille Université INSERM, IRD, SESSTIM, Sciences Economiques & Sociales de la Santé &Traitement de l’Information Médicale, Marseille, France; 4 ORS PACA, Observatoire régional de la santé Provence-Alpes-Côte d’Azur, Marseille, France; 5 Unité de Recherche Démographique (URD) Université, Lomé, Togo; 6 World Health Organization, Headquarters, Genève, Switzerland; 7 African Field Epidemiology Network (AFENET), Kampala, Uganda; 8 Institut supérieur des sciences de la population (ISSP), ISSP, Université de Ouagadougou, Ouagadougou, Burkina Faso

**Keywords:** Immunization, Children, Vaccine coverage, Togo

## Abstract

**Background:**

Incompleteness of vaccination coverage among children is a major public health concern because itcontinues to sustain a high prevalence of vaccine-preventable diseases in some countries. In Togo, very few data on the factors associated with incomplete vaccination coverage among children have been published. We determined the prevalence of incomplete immunization coverage in children aged one to five years in Togo and associated factors.

**Methods:**

This was a cross-sectional study using secondary data from the 2010 Multiple Indicator Cluster Surveys (MICS4) conducted in 2010 among children aged 1 to 5 years in Togo. This survey was conducted over a period of two months from September to November, 2010.

**Results:**

During Togo’sMICS4 survey, 2067 children met the inclusion criteria for our study. Female children accounted for 50.9 % (1051/2067) of the sample and 1372 (66.4 %) lived in rural areas. The majority of children (92.2 %; 1905/2067) lived with both parents and 30 % of the head of households interviewed were not schooled (620/2067). At the time of the survey, 36.2 % (750/2067) of the children had not received all vaccines recommended by Expanded Program on Immunization (EPI).

In multivariate analysis, factors associated with incompleteness of immunization at 1 year were: health region of residences (Maritime aOR = 0.650; *p* = 0.043; Savanes: aOR = 0.324; *p* <0.001), non-schooled mother (aOR = 1.725; *p* = 0.002),standard of living (poor: aOR = 1.668; *p* = 0.013; medium: aOR = 1.393; *p* = 0.090) and the following characteristics of the household heads: sex (aOR = 1.465; *p* = 0.034), marital status (aOR = 1.591; *p* = 0.032), education level(non-educated: aOR = 1.435; *p* = 0.027.

**Conclusion:**

The incomplete immunization coverage among children in Togo remains high. It is necessary to strengthen health promotion among the population in order to improve the use of immunization services that are essential to reduce morbidity and mortality among under five years old children.

## Background

Every year, vaccine preventable diseases are responsible for about 29 % of deaths among children under 5 years in the world [1]. In 2013, the total number of children who died from vaccine preventable diseases was estimated at 1,500,000. Of these, 13.3 % were related to haemophilus influenzae type B, 13.0 % to pertussis and 7.9 % to measles [1, 2]. However, 2–3 million child deaths of these deaths can be prevented through vaccination against diphtheria, tetanus, whooping cough and measles [1, 2]. Increasing immunization coverage is essential to reducing child mortality [1, 2]. Vaccination remains the best way to protect children under the age of five against such preventable diseases.

To ensure maximum protection of children, the World Health Organization (WHO) initiated and launched the Expanded Program on Immunization (EPI) in 1974 which was eventually adopted by all countries in the 1980s [3].

To date, this program covers 11 common childhood vaccine-preventable diseases in tropical Africa, including tuberculosis, diphtheria, pertussis, tetanus, poliomyelitis, measles, yellow fever, hepatitis B, infections due to *haemophilus influenzae* type B, pneumococcal conjugate vaccine and rotavirus vaccine. According to the immunization schedule in place, children before their first birthday should receive all the corresponding antigens to these diseases. But according to The United Nations Children’s Fund (UNICEF) estimates, one out of five new borns in the world do not receive one or more of the three doses of vaccines against diphtheria, tetanus and pertussis (DPT), which could be life-saving [1]. Despite coverage rates improving significantly in most countries since 2000, it is clear that immunization rates continue to be low in some African countries. For example, coverage with the first dose against measles in WHO’s AFRO region increased from 57 % in 2001 to 73 % in 2008 [4] but below the regional target of 80 %. However, these overall coverage rates hide many disparities between WHO regions, countries and even within a country [5].

In 2003, Burkina Faso [6] reported that its highest regional coverage was 72 % in the Central region while the lowest was 31 % in the Sahel region. Togo demonstrated similar results in 2010, with the Savanes region recording the highest complete vaccine coverage estimated at 65.4 % in contrast to the Lomé-commune region’s being lowest at 31.4 % [7].

This incompleteness of Immunization coverage among children is now a major public health concern because it continues to sustain a high prevalence of vaccine-preventable diseases and has undermined the achievement of the 4^th^ Millennium Development Goal of reducing under five children mortality rates by two-thirds between 1990 and 2015.

Several studies have been conducted in countries with limited resources to understand the various factors explaining the incompleteness of vaccine coverage. Most of these studies suggest that incompleteness of vaccine coverage was not only strongly associated with socio-economic characteristics of the population, but mainly to factors related to the organization and functioning of health systems in the country [5, 8–13]. Particularly in Togo, very few data on the predictors of incomplete immunization coverage among children have been published.

In this study we aimed at assessing the incompleteness of immunization coverage among children aged 1 to 5 years in Togo and determining the associated factors.

## Methods

### Study design and sampling

This was a cross-sectional study that involved review of secondary data of sampled children aged 12 to 59 months. The data were obtained from the Multiple Indicator Cluster Survey (MICS4) of Togo completed in 2010 [7]. This was a nationwide survey which involved 6039 households that included 6367 women, 1925 men (aged 15 to 49 years old) and 4746 children less than 5 years old.

The MICS 4 survey was cross-sectional and had a representative sample at national, health region and residence level. The six health regions were stratified by urban and rural areas. A total of 12 strata were formed and in each stratum, enumeration areas were selected. In each enumeration area, 15 households were selected in the list of all households. A total of 465 strata were selected of which 169 were in urban areas and 296 in rural areas [7].

### Eligibility criteria

We excluded children under 12 months, because theoretically they had not yet completed their immunizations (EPI routine vaccines) and children with no immunization card or record because the information collected from the mother could not be verified. The study then focused exhaustively on 2067 children aged 12 to 59 months.

### Study variables

In this study, vaccine incompleteness at the first birth day of the child (incomp_vacc) was our dependent variable. By the standards of WHO and UNICEF, as well as those of the EPI in Togo, a child is considered incompletely vaccinated at 1 year if he has not received all the routine EPI vaccines before his/her first birthday. Thus, vaccine incompleteness at 1 year was grouped into two categories: (1) for “vaccination incompleteness” and (0) for vaccination completeness”.

### Data collection

Data collection which took two months (September 6 to November 4, 2010) was conducted by interviewers who received specific training from 9 to 28 August 2010.

Four types of questionnaires were used as tools for collecting data from the MICS 4: (1) a household questionnaire to collect information on household members and housing characteristics, (2) an individual men’s questionnaire administered to male members of the household 15 to 49 old, (3) individual women's questionnaire administered to female members of the household 15 to 49 old, and (4) an individual questionnaire administered to mothers or caretakers of all children living in the household. The data used for our study was mainly from individual questionnaires administrated to mothers or care takers of children when mothers were absent.

### Data analysis

Data analysis including descriptive analysis (univariate) and explanatory analysis (multivariate) were conducted using the SPSS statistical software (Statistical Package for Social Science) Version 21.

For continuous variables, means and standard deviations were calculated while for categorical variables we calculated proportions. Our primary outcome of interest was children who had not completed vaccination at 1 year compare to those who had complete their vaccination.

Pearson chi-square test or Fisher exact test were used when appropriate in bivariate analysis.

Multivariate backwards stepwise logistic regression analysis was performed to identify independent risk factors for the dichotomous outcome “incompleteness of vaccination coverage at 1 year”. All variables significant during the bivariate analysis at a *p*-value less than 0.2 were included in the multivariate analysis to assess the adjusted effect and derive the adjusted odds ratio (aOR) of each on the primary outcome variable. We allocated the value “1” to the dependent dichotomous variable if the child has incompleteness vaccine coverage and the value “0” otherwise. A 95 % level of confidence was applied throughout.

## Results

### Baseline characteristics

Of the 4746 children surveyed during the MICS4 in Togo, 2067 met the inclusion criteria for our study. Female children accounted for 50.8 % (1051/2067) of the sample and 1372 (66.4 %) lived in rural areas. The majority of children (92.2 %, 1905/2067) lived with both parents. In 30 % of households, the household heads were illiterate (620/2067). In addition, 50 % (1033/2067) of the household heads were Christian and 87.1 % were men (1802/2067). The Plateaux region had the highest number of respondents (23.6 %, 488/2067) followed by Maritime region (22.9 %, 474/2067), Savanes region (18.4 %, 380/2067), Kara region (13.3 %, 274/2067), Lome-Commune region (11.9 %, 247/2067) and Centrale region (9.8 %, 203/2067).

### Vaccination schedule in Togo

The number of children vaccinated by age in Togo peaked between the age of birth to 11 weeks when BCGis predominantly administered at birth. However, many vaccines were administered both before and after the observed vaccine peaks. Overall, BCG-vaccine was the most administered vaccine followed by DPT1,DPT2 and measles vaccine. DPT3 was the most poorly vaccine administered.

### Immunization coverage in Togo

In the study sample, 36.2% (750/2067) of children had not received one or more of the vaccines recommended by the EPI at the time of the survey. This rate ranged from 23.5% in the Savanes region, to 45% in the Plateaux region.

After controlling for confounding through multiple logistic regression model, children aged 1 to 5 years living in Savanes (aOR = 0.32; *p* <0.001) and Maritime region (aOR = 0.65; *p* = 0.04) were less likely to be incompletely vaccinated compared to those living in the Lomé-Commune region.

Households headed by men (*p* = 0.03) and single mothers (*p* = 0.03) were 1.5 and 1.6 times respectively more likely to be incompletely vaccinated. Illiterate mothers were 1.7 times more likely to be poorly vaccinated compared to those with secondary education level and above (Table 1). Children from Christian households were less likely to be incompletely vaccinated compared to Muslims (aOR = 0.54; *p* = 0.01). Children from poor households were 1.7 more likely to be poorly vaccinated (*p* = 0.01) (Table 1).

**Table 1:** Predictors of incomplete immunization among children aged 12 to 59 months old in Togo, 2010

	Bivariate		Multivariate	
	Incomplete immunization (Yes)	*p*-value (khi-2)	aOR	95 % CI
Gender of the child		0.831		
Male (*n* = 1016)	371 (36.5 %)		0.986	[0,799 - 1.217]
Female (*n* = 1051)	378 (36.0 %)		1	-
Gender of household head		0.306		
Male (*n* = 1802)	662 (36.7 %)		1.465	[1,030 - 2.083]
Female (*n* = 265)	88 (33.2 %)		1	-
Living in couple (*N* = 2067)		**0.087**		
Yes (*n* = 1905)	678 (35.6 %)		1	-
No (*n* = 141)	61 (43.2 %)		1.591	[1,042 - 2.429]
Level of education of the mother (*N* = 2067)		**<0.001**		
None (*n* = 848)	355 (41.9 %)		1.725	[1,220 - 2.439]
Primary (*n* = 802)	292 (36.4 %)		1.295	[0,948 - 1.769]
Secondary and upper (*n* = 417)	105 (25.2 %)		1	-
Level of education of the head of household (*N* = 2067)		**<0.001**		
None (*n* = 620)	248 (40.0 %)		1.435	[1,043 - 1.975]
Primary (*n* = 656)	286 (43.6 %)		1.606	[1,231 - 2.095]
Secondary and upper(*n* = 770)	217 (28.2 %)		1	-
Religion of the head of household (*N* = 2067)		**<0.001**		
Muslim (*n* = 293)	115 (39.2 %)		1	
Christian (*n* = 1033)	325 (31.5 %)		0.637	[0,45 0 - 0.902]
Animist(*n* = 739)	308 (41.7 %)		0.850	[0,594 - 1.216]
Ethnic group of the head of household (*N* = 2067)		**0.002**		
Adja-Ewé (*n* = 764)	306 (40.0 %)		1	-
Kabyè-Tem (*n* = 594)	210 (35.3 %)		0.541	[0,374 - 0.781]
Para-Gourma (*n* = 477)	137 (28.7 %)		0.623	[0,380 - 1.022]
Other(*n* = 219)	89 (40.6 %)		0.781	[0,534 - 1.141]
Standard of living (*N* = 2067)		**<0.001**		
Poor (*n* = 891)	377 (42.3 %)		1	
Medium (*n* = 434)	164 (37.8 %)		1.668	[1,115 - 2.496]
Rich(*n* = 742)	212 (28.6 %)		1.393	[0,950 - 2.042]
Place of residence		**<0.001**		
Urban (*n* = 695)	208 (30.0 %)		1	-
Rural (*n* = 1372)	542 (39.5 %)		0.975	[0,671 - 1.416]
Health region		**<0.001**		
Lomé-Commune (*n* = 247)	88 (35.6 %)		1	-
Maritime (*n* = 474)	156 (32.9 %)		0.650	[0,429 - 0.986]
Plateaux (*n* = 488)	220 (45.0 %)		1.059	[0,674 - 1.663]
Central (*n* = 203)	89 (43.8 %)		1.138	[0,651 - 1.992]
Kara (*n* = 274)	103 (37.6 %)		1.096	[0,632 - 1.900]
Savanes (*n* = 380)	89 (23.5 %)		0.324	[0,173 - 0.607]

## Discussion

We assessed the factors leading to incomplete immunization of children under 5 years in Togo. The study noted that 36.3 % of children were incompletely vaccinated at 1 year. Factors that were associated with incompletely vaccination coverage were; place of residence, gender of the head of household, living in couple, level of education of the mother, religion and standard of living.

In our study, incompleteness of immunization coverage was reported at 36.3 % of children aged one to five. Higher rates (49.8 %) were reported in Burkina Faso in a similar study conducted in rural and semi-urban communities [10], in Ethiopia (74.4 %) in a study co nducted in the district of Jigjiga [13] and in Pakistan (66 %) [14]. This difference could be explained by the effort of the ministry of health to implement the strategy “reach every district” with the support of partner civil society. However, the rate of incomplete immunization of under 5 year children remains high in Togo and that points out a major concern regarding the fight against vaccine-preventable diseases which cause a large proportion of child deaths, despite the knowledge that a vaccination coverage rate of at least 95 % is required to control these diseases [11]. This proportion of incomplete immunization coverage hardly tend to decrease in Togo, indeed a recent study showed that 30 % of the children did not complete their immunization [15].

Likewise in Mali [16], and Burkina Faso [6], incompleteness of immunization coverage rate in Togo was ranged from 23.5 % in the Savanes region to 45 % in the Plateaux region; this implies that the vaccine coverage rate at national level hides huge disparities between the health regions. This regional disparity can be explained both by socio-cultural factors, for example, the north of Togo is known as somehow influenced by culture from Nigeria [11]. Similarly this was also observed by Ruijs et al. in Netherlands where the social characteristics of the populations were reported to be essentially the explanation of regional vaccine coverage disparities [17].

In addition to socio-cultural factors, regional differences in vaccine incompleteness coverage in Togo could also been explained by strategic choices favoring immunization in certain regions (for example, Savanes and Maritime) where financial partners have invested substantially in the improvement of primary health care compared to other regions (eg. Lomé-commune). The high rate of incomplete vaccine coverage could be explained by the influence of religion and traditional culture on people to refuse vaccination. In the two regions of the country, outreach activities and other primary health care activities continue to be financed by foreign financial partners and is therefore very well implemented [18]. In these regions, immunization services are brought closer to the populations thus facilitating their use.

We also found that gender of the head of the household was significantly associated with vaccination incompleteness. The data for Togo suggested that children of households headed by women were more likely to be vaccinated in contrast, data from Jamaica and Trinidad and Tobago reported that children of households headed by women were poorly vaccinated due low economic power [19]. The probable explanation in our study is that women are more sensitive and more concerned about their child’s health status than men; hence it’s needed to improve the decision-making ability and economic power of women in many African communities where the decision to use health services is entirely devoted to men [20]. Mothers play a very important role in their children immunization. Indeed, in prior studies in Ethiopia [21] and Nigeria [22], the authors reported that women’s participation in decision-making in the household and especially the improvement of its economic power improved immunization coverage of children.

Children living in a family home with both parents are more likely to complete immunization coverage. This finding is consistent with the results reported by Cherkaoui et al. in the High Atlas region of Morocco [23].

Our study also supports the notion that economic conditions influence the incompleteness of immunization coverage in children. This is probably because access to immunization services can be affected by indirect costs linked to vaccination such as the purchase of immunization records cards, transport cost or medication for vaccine-related care. This has been suggested in studies conducted in rural communities in Burkina Faso [9] and in Nairobi, Kenya [24].

Education level of the head of household also favors immunization levels. It was noted that the higher the education level of the mother, the more likely they are to complete the child’s immunization schedule. This result is consistent with those obtained in Mozambique [8], Nigeria [22] in Burkina Faso [12] and Ethiopia [13]. Indeed, education of a woman clearly affects her living standards and accessibility to information on the use of health services in general and in particular, immunization services. Moreover, being educated facilitates a woman’s communication with health personnel, thus, ensuring better understanding and reception of information on practices that have a positive impact on the well-being of children.

Lastly, ethnicity may enhance certain cultural values that could influence perceptions and health behaviors. Our study shows that children in households headed by members of the Adja-Ewe ethnic group had very low coverage compared to the Para-gourma and Kabyè-tem ethnic groups. This could be explained by the fact that the Adja-Ewe, originating in and forming the majority of southern Togo population are mostly followers of “voodoo”, a traditional religion that does not easily adhere to modern medicine and frequently attributes vaccine-preventable diseases to supernatural causes [25]. Mutua et al. [24] in a study in Nairobi, Kenya also reported a statistically significant effect of ethnic groups on the incompleteness of immunization coverage.

### Limitations of the study

Analyzing data only on living children and those who had immunization records cards and excluding information on dead children or those without immunization cards may have precluded a better assessment of the risk of vaccine incompleteness. The second limitation was the unavailability of data related to the provision of care. In order to better understand regional differences in vaccine coverage, it would have been necessary to consider the characteristics of the health facilities in addition to those of populations. The study sample was obtained from a stratified sampling design and the statistical analysis were performed without considering the weight of individuals, it would be biased to generalize these results to all children of the same age of Togo.

## Conclusion

With 36.3 % of children under five not being fully vaccinated as per the EPI vaccines schedule, the prevalence of incomplete immunization coverage among children remains high in the study sample. Our study identified the different factors influencing this prevalence including gender of the household head, marital status, level of parents’ education, standard of living, ethnicity and residing in certain health regions. It is therefore important to strengthen health education among the population and to improve the provision of EPI activities at peripheral health facilities especially when the country is willing to introduce new vaccines in the EPI to reduce morbidity and mortality due to vaccine preventable diseases among under 5 years old children.

## Abbreviations

aOR: Adjusted odd ratio
DPT: Diphtheria, tetanus and pertussis
EPI: Expanded program on immunization
MICS4: Multiple indicator cluster surveys 4
UNICEF: The United Nations Children’s Fund
WHO: World Health Organization
